# A Skin-Inspired Self-Adaptive System for Temperature Control During Dynamic Wound Healing

**DOI:** 10.1007/s40820-024-01345-0

**Published:** 2024-03-11

**Authors:** Yaqi Geng, Guoyin Chen, Ran Cao, Hongmei Dai, Zexu Hu, Senlong Yu, Le Wang, Liping Zhu, Hengxue Xiang, Meifang Zhu

**Affiliations:** State Key Laboratory for Modification of Chemical Fibers and Polymer Materials, College of Materials Science and Engineering, Donghua University, 2999 North Renmin Road, Shanghai, 201620 People’s Republic of China

**Keywords:** Thermo-reception, Self-regulation, Flexible electronic system, Wound healing

## Abstract

The thermoregulating function of skin that is capable of maintaining body temperature within a thermostatic state is critical. However, patients suffering from skin damage are struggling with the surrounding scene and situational awareness. Here, we report an interactive self-regulation electronic system by mimicking the human thermos-reception system. The skin-inspired self-adaptive system is composed of two highly sensitive thermistors (thermal-response composite materials), and a low-power temperature control unit (Laser-induced graphene array). The biomimetic skin can realize self-adjusting in the range of 35–42 °C, which is around physiological temperature. This thermoregulation system also contributed to skin barrier formation and wound healing. Across wound models, the treatment group healed ~ 10% more rapidly compared with the control group, and showed reduced inflammation, thus enhancing skin tissue regeneration. The skin-inspired self-adaptive system holds substantial promise for next-generation robotic and medical devices.

**Supplementary Information:**

The online version contains supplementary material available at 10.1007/s40820-024-01345-0.

## Highlights

- An interactive electronic system inspired by the temperature self-regulation of human skin.
- Heat stimulation therapy and temperature monitoring during dynamic wound healing.
- Mechanism of temperature self-regulation during dynamic wound healing.

**Supplementary Information:**

The online version contains supplementary material available at 10.1007/s40820-024-01345-0.

## Graphical abstract

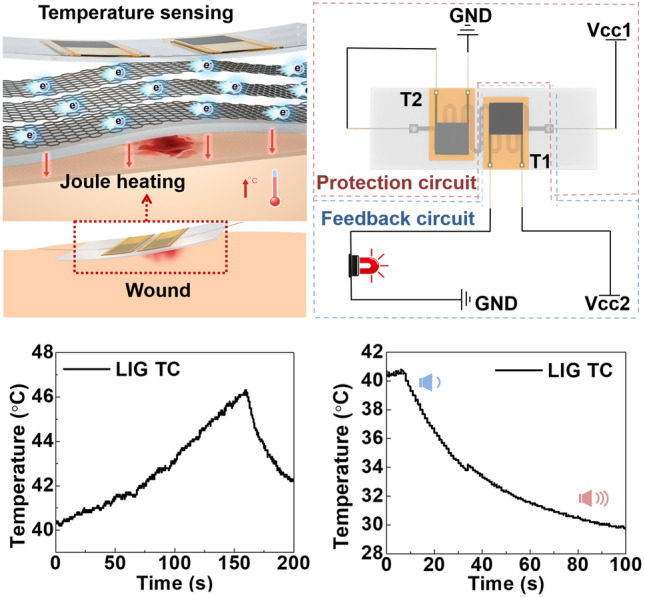

## Introduction

As all know, there is a complex sensor system within the skin, enabling it to perceive the surrounding environment, such as temperature, pressure, strain, and vibration [1–3]. Among these functions, regulation of temperature is one of the key functions for homo-thermal animals during their lifecycle. As for its temperature control mechanism, thermo-sensitive sensory neurons embedded in the skin will first sense the temperature changes, then they will convey thermal cues from the periphery to the central nervous system [4–7]. Finally, our brain will trigger a series of accommodative responses to achieve temperature regulation. For example, enhancing heat dissipation in a warm environment or suppressing heat dissipation in a cold environment by controlling the relaxation/contraction of capillaries [8, 9]. However, skin damage is inevitable in daily life, which will not only cause local inflammation and infection, even lead to lost skin sensory and regulatory functions [10]. In addition, it was reported that the temperature around the wound tends to be slightly higher because the blood flow rate at the damaged area increased [11]. Mild thermal stimulation has been shown to positively help wound repair, but most hyperthermia devices do not have thermal feedback, often causing additional side effects and pain for patients, such as skin burns and inflammation [12–14]. Thus, it is of great significance to rebuilding temperature control of the skin for people who have skin damage, especially for its dynamic wound healing.

The use of electronic devices to reconstruct the functions of the damaged skin have profound implications in the field of bionic robots and mechanical prosthetics, especially restoring the thermal sensing and adaptive regulation functions can greatly improve the life quality for people with skin injuries [15, 16]. While electronic skin thermo-sensing has been used for bionic skin applications, but most of them lack self-regulation function which is important to users and can greatly improve comfort [17–19].

As reported previously, temperature sensing usually relies on resistance thermometers and conductive composites of electronic skin (e-skin) [20–23]. The former one usually has a linear temperature coefficient of resistance (TCR), which can continuously monitor skin temperature with high precision, but the sensitivity is limited [24, 25]. As for the other mechanism of temperature response is the thermal expansion of conductive polymer composites [26–28]. Tomoyuki Yokota et al. doped graphite in a semi-crystalline polymer to produce an ultra-sensitive and flexible temperature sensor [29]. This method is universal and strain insensitive, and the sensitivity is improved by adjusting the difference of thermal expansion coefficient between the conductive particle and the polymer matrix.

Unfortunately, currently reported electronic skin usually lacks the function of the body’s dynamic temperature regulation. Mufang Li et al. introduced thermoregulation into electronic skin for the first time, preparing a polyolefin elastomer nanofiber film that is transparent to infrared radiation, through which body heat can be dissipated to achieve thermal regulation properties [30]. It successfully increased the heat dissipation of the skin, while still difficult to realize the dynamic adjustment of temperature. Phase change materials (PCMs) have a similar function of dynamic temperature regulation, which can adsorb heat at high temperature and release heat at low temperature. Nevertheless, the temperature regulation ability of PCMs is limited in uncontrollable ambient temperature [31, 32]. In order to further simulate the constant temperature and self-regulation characteristics of the skin, the heater and temperature sensor are constructed by using the metal nanonetwork, which can adjust and monitor the temperature in real time. However, the temperature monitoring sensor cannot generate signals that directly act on the heater to make its temperature change accordingly [33, 34]. Moreover, for people with skin defects, it is significant to realize the dynamic temperature regulation during wound repair. Therefore, a bionic intelligent electronic device that integrate the features of thermos-sensing, self-regulation and biocompatibility is highly desired. In the perspective of mass production and preparation, it is also necessary to have the advantages of simple process and low cost. Here, inspired from the temperature controlling system of skin, we design an interactive electronic system that imitates the skin thermo-regulation function. More importantly, this system can promote tissue repair in wounded skin. The system is consisted of two main units, one is a laser-induced graphene (LIG) array as the temperature control unit, and another is two highly sensitive flexible Positive Temperature Coefficient (PTC) thermistors (the temperature regulating unit). When the temperature of the electronic skin is below 33 °C, the thermistor that controls the low temperature can activate the buzzer alarm. Once the temperature is above 46 °C, the thermistor that controls the high temperature can respond within 10 s and bring the temperature down. More importantly, the self-thermal regulation of the e-skin could adjust the wound area in a suitable temperature range thus enhance wound regeneration (the healing rate is increased by about 10%). The highly perceptive, self-regulating, multifunctional electronic system offers promising applications in the field of bionic skin electronics and personalized medical treatment.

## Experimental Section

### Chemicals and Materials

Octadecyl acrylate (AR, 99%) and butyl acrylate (AR, 99%) were purchased from RHAWN, Octadecyl acrylate (OA) without treated, but the polymer inhibitor was removed before butyl acrylate (BA) was used. The initiator 2,2ʹ-Azobis(2-methylpropionitrile) (AIBN) (99%) that recrystallization was purchased from MACKLIN. Two solvents, N,N-Dimethylformamide (DMF) (AR, 99.5%) and tetrahydrofuran (THF) (AR, 99%), were purchased from RHAWN and ALADDIN, respectively. Carbon black (CB) was purchased from ALFA. Dimethylsiloxane (PDMS) was purchased from DOW CORNING. Styrene Ethylene Butylene Styrene (SEBS) was purchased from Kraton, USA.

### Preparation of LIG TC

The pre-designed snake pattern was led into the software CorelDRAW, and the 25 µm PI film was carved using the Laser Pro C180 II laser engraving machine. To make the PI base flat, it is fixed on a hard plastic board. Set scanning parameters: horizontal scanning, speed 5 cm^2^ h^−1^, power 25 w, DPI 500, PPI 400. The PDMS main agent and curing agent were mixed at a ratio of 10:1. After vacuum defoaming, the PDMS was coated on the carved pattern and dried in an oven at 70 °C for 2 h. Then tear off the cured PDMS and transfer the multi-layer graphene structure. Fix the wire at both ends of the serpentine pattern with silver paste, and then dry in an oven at 70 °C for 30 min. The PDMS paste was prepared and dried again for encapsulation.

### Preparation of PTC Thermistors

The Acrylate copolymer (AC) was mixed with SEBS at a ratio of 7/3 (the minimum content of SEBS added to toughen the composite, as shown in Fig. S4), and then dissolved in THF (solid content 10%), followed by adding 10% CB (critical seepage threshold concentration, as shown in Fig. S15), and dispersed by ultrasonic for 1 h (water bath temperature less than 30 °C). Then drop the PTC ink into the gold-plated interfinger electrode sheet (electrode sheet 12 µm thick, 15 pairs of interfinger, 60 µm line width and line distance). Drying at room temperature to obtain PTC thermistors with a material thickness of about 50 µm, as shown in Fig. S16.

### Assembly of the TRES

The PTC thermistors are placed on the LIG TC, sealed, and fixed with waterproof PU tape, and the interactive thermoregulation Bionic electronic system (TRES) is prepared.

### Characterization and Evaluation of Materials

The microstructures of LIG TC and PTC thermistors were observed by scanning electron microscope (SU8010). Elemental analysis of LIG was performed using an X-ray photoelectron spectrometer (ESCALAB250XI) and a Fourier infrared spectrometer (Nicolet6700). The degree of graphitization of LIG was analyzed using a Raman spectrograph (in Via-Reflex). An in situ electrochemical test X-ray diffractometer (D8 ADVANCE) was used to analyze the crystal patterns of LIG. The melting and crystallization of PTC thermistors were characterized using a differential scanning calorimeter (METTLER DSC1). The thickness of the PTC thermistor was measured using a step profiler (Dektak XT). Thermal expansion coefficients of PDMS and PI were tested using a thermo-mechanical analyzer (TMA 402F3). The electrical performance of the device was tested using two digital source meters (Keithley 2450). The temperature response performance of the device was tested using a digital multimeter (Keithley DMM 6500) and a hot and cold station (INSTEC mK2000). Use an oscilloscope (Tektronix MSO44) to record the electrical output. The thermal infrared imager (FOTRIC 220 USA) was used for real-time temperature monitoring.

### Animal Experiment

BALB/c mice, purchased from BiKEyi Biotechnology Co., LTD. (License No. SCXK (Shanghai) 2018–0006; Certificate No. 20180006045427), 18, SPF grade (Shanghai Laboratory Animal Quality Supervision and Inspection Station evaluation), male, body mass 18–20 g. All the animals were reared in the animal laboratory for 3–5 days with 12/12 h circadian rhythm and 35% humidity. 18 mice were ear-tagged before modeling. Mice were anesthetized by intramuscular injection of Sutai®50 at a dose of 1.5 mL kg^−1^ bw^−1^ and fixed in the prone position. The skin of the operation area was prepared and disinfected with alcohol and iodine. A round, full-layer skin lesion with a diameter of 5 mm caused by drilling. The mice were randomly divided into six groups (3 mice per group): control group *2, LIG heating group *2, and commercial heating group *2. As a control, the wounds of the two heating groups were completely covered by the heater, and the temperature was controlled by voltage (DC power supply) for 30 min a day. Heat for 15 min at a time, with 20-min intervals, for 3 consecutive days (days 1–3). Wound photos were taken on days 0, 3, 8, and 12 in all groups. Wound area was measured on photographs using ImageJ image analysis software (National Institutes of Health) to assess the extent of wound healing, the wound area was analyzed by tracking wound edges, and the wound healing rate was calculated by the following formula:

1 $$W_{{{\text{healing}}\;{\text{rate}}}} = {\raise0.7ex\hbox{${\left( {W_{{0\;{\text{day}}}} - W_{{t\;{\text{day}}}} } \right)}$} \!\mathord{\left/ {\vphantom {{\left( {W_{{0\;{\text{day}}}} - W_{{t\;{\text{day}}}} } \right)} {W_{{0\;{\text{day}}}} }}}\right.\kern-0pt} \!\lower0.7ex\hbox{${W_{{0 {\text{day}}}} }$}}$$

Here, *W*_0 day_ is the wound area of D_0_, in mm^2^, *W*_*t* day_ is the area of the wound on day *t*, in mm^2^, *t* = 3, 5, 8 and 12. *W*_(healing rate)_ stands for wound healing rate in mice.

One representative fixed sample was selected from each group. After paraffin embedding, the whole layer was cut in the center of the defect, and histological analysis was performed by H&E staining and Masson staining.

### Statistical Analysis

SPSS 26.0 software was used for statistical analysis of the data. The measurement data were tested for normal distribution and homogeneity of variance. The measurement data conforming to normal distribution were presented as mean ± standard deviation ($\overline{x }$ ± s), the homogeneity of variance was tested by the Tukey test, and the inconsistency of variance was tested by the Tamhane T2 test. Mann–Whitney test was used for measurement data with non-normal distribution. In *p* < 0.05 was considered statistically significant.

## Results and Discussion

### Design Concepts and System Features

**Fig. 1:**
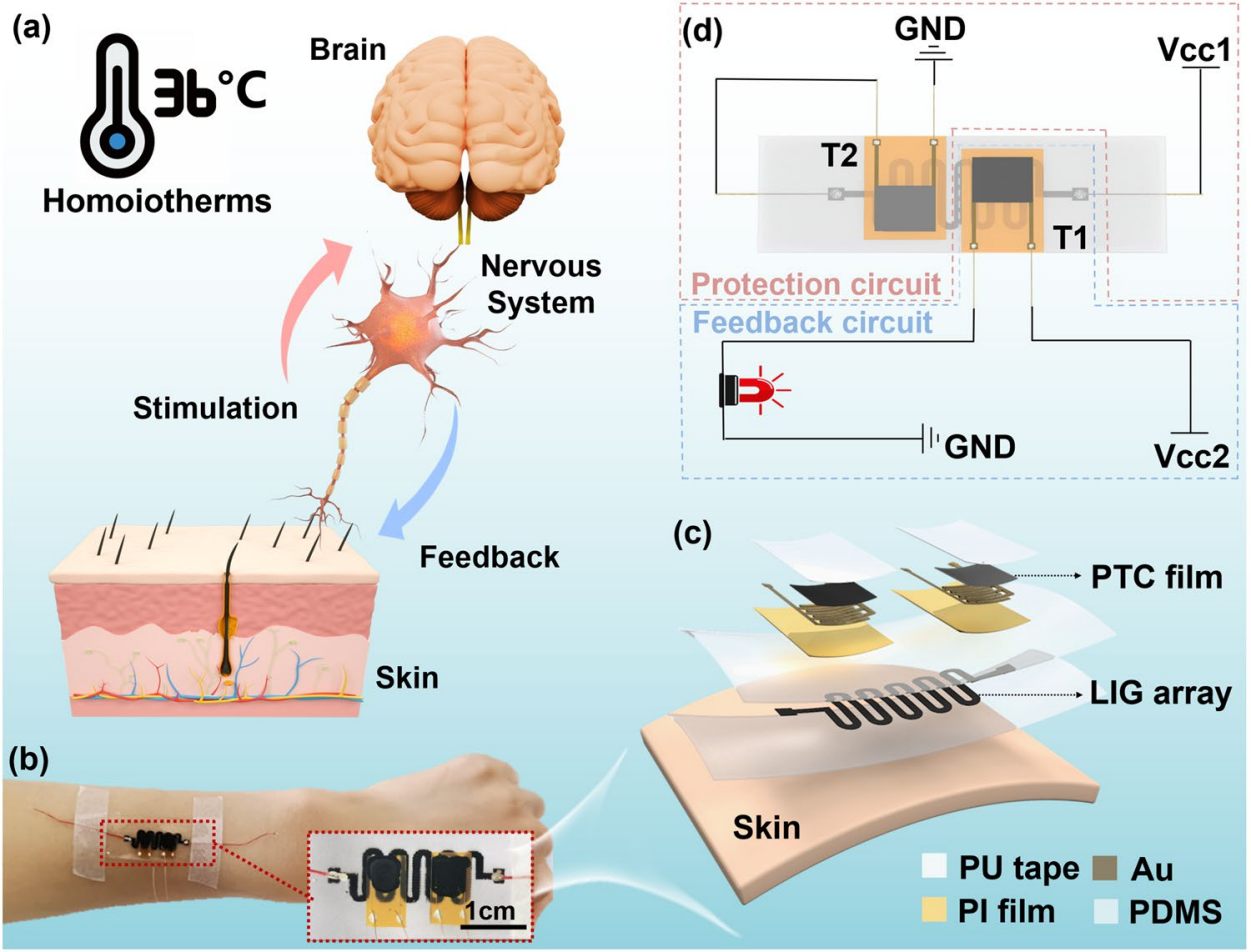
Design concepts and system features of the interactive thermo-regulation bionic electronic system (TRES). **a** Temperature self-regulation mechanism in homoiotherms. **b** The actual picture of TRES placed on the arm. **c** Temperature self-regulation mechanism of TRES. **d** Assembly diagram of TRES

As described above, the temperature tunability of skin relies on a sensing and feedback process, inspired by this process, we designed an interactive thermoregulation bionic electronic system (TRES), as shown in Fig. 1a. The device mainly consisted of two components, one is thermal controlling unit and another is temperature monitoring unit. The thermal controlling unit is prepared with a graphene array (LIG) encapsulated with PDMS. The LIG possesses a joule heating effect, is put at the bottom layer of the TRES, which can contact with tissues directly. As for the temperature tuning unit, two polymer-based PTC thermistors with designable switching temperatures are used. The PTC thermistors can monitor the substrate temperature in real-time and give feedback when the temperature is out of the chosen temperature range. Finally, the top of the device, a layer of polyurethane (PU) tape is used to sealed system, allowing the fixation between temperature tuning unit and the thermal controlling unit, then the TRES can be holistically attached to the skin, as shown in Fig. 1b, c. The principle of the bionic self-regulating system is shown in Fig. 1d, which is divided into an external feedback circuit and an internal protection circuit. In a normal working state, the LIG thermal control unit (TC) is maintained at normal body surface temperature, and thermistor 1 (T1) is in a high resistance state and thermistor 2 (T2) is in a low resistance state. When the voltage (Vcc1) is unloaded, the temperature of the TC drops, and the resistance of T1 decreases rapidly, activating the alarm. When the Vcc1 increases, the temperature exceeds the switching temperature of T2 making the resistance rise, while the circuit current decreases, and the temperature of the TC decreases.

### Preparation and Joule Thermal Properties of LIG TC

**Fig. 2:**
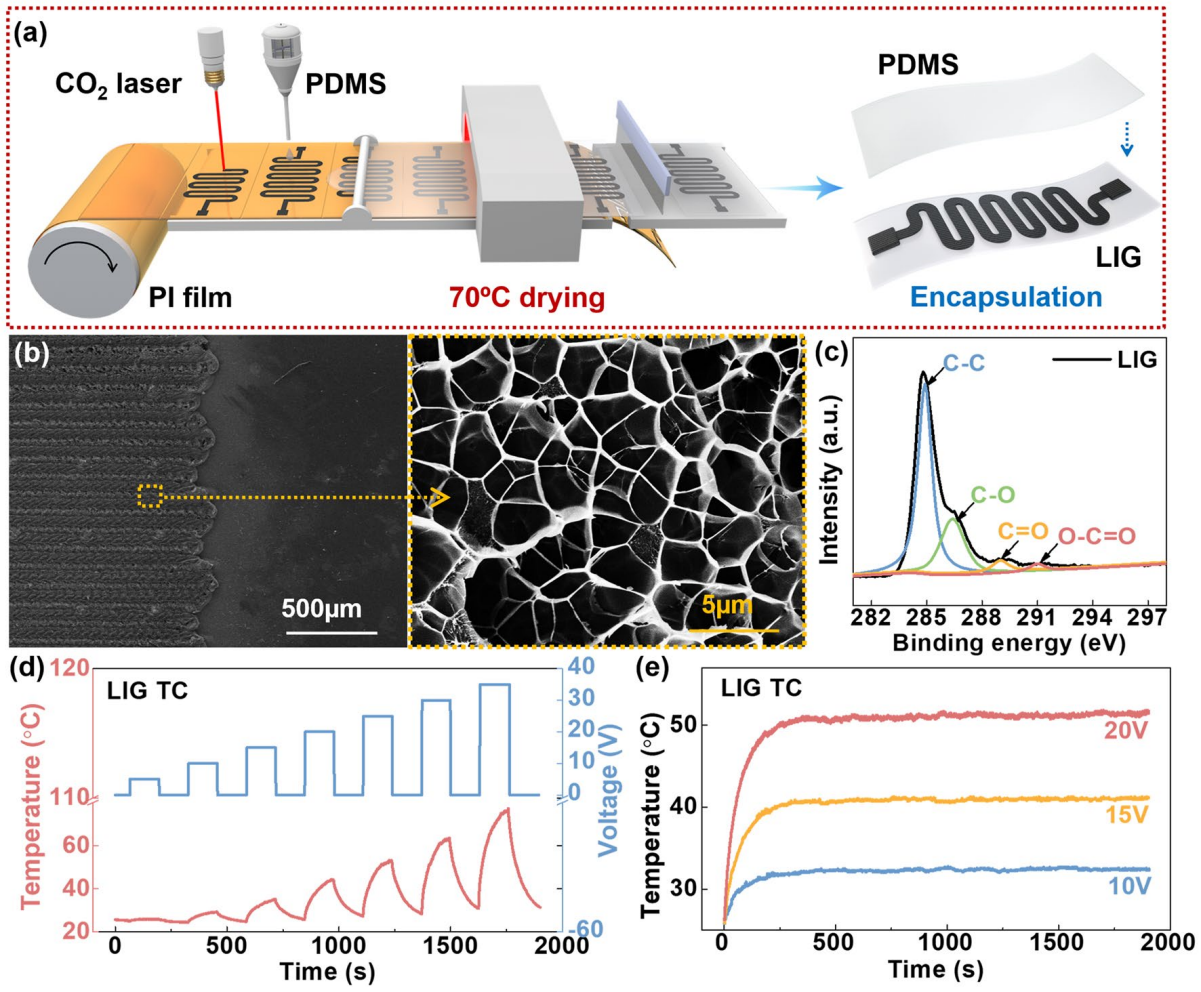
Preparation and Joule thermal properties of the LIG TC. **a** Preparation process of the LIG TC. **b** SEM image of LIG (texture structure and porous structure). **c** XPS analysis of LIG. **d** Joule thermal properties LIG TC. **e** The heating stability of the LIG TC

To achieve rapid temperature control, LIG was prepared as a temperature control unit (Fig. 2a). Commercial polyimide (PI) film is chosen as the source materials for forming high-quality LIG due to their thermomechanical stability and rich carbon content [35, 36]. Next, a CO_2_ laser was used to convert PI into LIG structures effectively along a pre-designed serpentine pattern. The serpentine graphene layer was then transferred to a PDMS substrate and further encapsulated by PDMS. This selective, low-cost, chemical-free and environmentally friendly patterning technology has a broad application prospect for industrial production [37].

As shown in Fig. 2b, the obtained LIG has a neat texture structure and a disordered porous structure, which was due to that at the direction of the CO_2_ laser transverse scanning, gas will form in the carbonization process, thus resulting in a honeycomb porous structure [38, 39]. The result of X-ray diffraction (XRD) (Fig. S1a) shows that LIG has a strong diffraction peak around 26°, which is a typical (002) lattice plane of graphite. Besides, the diffraction peak around 43° belongs to the (100) crystal face of graphene. The graphitization degree of LIG can be calculated from the Raman spectroscopy test. As Fig. S1b shows, the Raman characteristic peaks of LIG are at 1350, 1580, and 2700 cm^−1^, corresponding to the D-peak, G-peak, and 2D peak of graphene, respectively. The *I*_D_/*I*_G_ ratio (peak area) is calculated as 0.48, which proves that a high-quality multilayer graphene structure was produced by the laser engraving. To further prove the structure of LIG, an X-ray photoelectron spectroscopy (XPS) test was carried out. The result in Fig. 2c shows that functional groups “C–C” and “C–O” are mainly present in LIG, and the content of “C=O” and “O–C=O” is very low. Fourier Transform Infrared Spectroscopy (FTIR) diagram Fig. S1c also confirms the existence of these functional groups, and the multilayer graphene structure is successfully prepared [40, 41]. In order to verify whether there is LIG residue in the stripped substrate, Raman characterization was performed on the PI substrate after stripping LIG. As shown in Fig. S2, no graphene characteristic peaks were found, demonstrating that the LIG layer was stripped off the PI substrate.

To verify the Joule thermal performance of LIG TC, we used a digital source meter (Keithley2450) to input pulse voltage from 0 to 35 V (belong to human safety voltage (< 36 V), and used a thermal infrared imager to monitor the temperature of LIG TC during heating and cooling process. We first compared the joule heat of the LIG on PI substrate and encapsulated with PDMS, as shown in Fig. S3. Before stripping, the heat is mainly concentrated on the LIG electrode (Fig. S3b). After stripping and being packaged by PDMS, a part of the heat begins to conduct in the horizontal direction, forming a more uniform heating field (Fig. S3a). The size and thickness of LIG electrode and PDMS layer are shown in Fig. S4. It can be seen from Fig. 2d that the temperature rises with the increase of voltage. Besides, according to Joule’s law, the temperature (*T*) is proportional to the square of the voltage (U^2^). When the voltage is added to the maximum measured voltage of 35 V, the temperature of the LIG TC can reach 80 °C. As shown in Fig. S5, the current through LIG TC increases with the impressed voltage, and its resistance is linearly related to temperature, making it easy to calculate the temperature according to the resistance value. In terms of promoting wound healing, mild thermal stimulation such as around 40 °C is suitable, when the power of LIG TC is about 0.15 W, and the current (4 mA) is lower that the human safety current of 10 mA. In contrast, the commercial heater is composed of polyimide (PI) as the substrate and metal circuit as the heating element, the preparation conditions usually need high vacuum conditions. The basic electric heating properties of the commercial heater are shown in Fig. S6. It can be seen that the current of the commercial heater (70 mA) far exceeds the safe current of the human body at 40 °C. In fact, due to the high thermal conductivity of graphene, the heat loss during heating can be reduced compared to traditional heater. Subsequently, we tested the response stability of the LIG TC, the result was shown in Fig. 2e. When 10, 15, and 20 V voltages were applied to the LIG TC, it can be monitored that the temperatures reached 30, 40, and 50 °C, respectively. And all the response time of three different group is less than 250 s, while the temperature is kept constant for more than half an hour during the test. Then we tested the thermal cycling performance of LIG TC, as shown in Fig. S7. When applying the same pulse voltage, the generated temperature remained stable, indicating that LIG TC has heating stability.

### Preparation and Thermo-Response Properties of PTC Thermistors

**Fig. 3:**
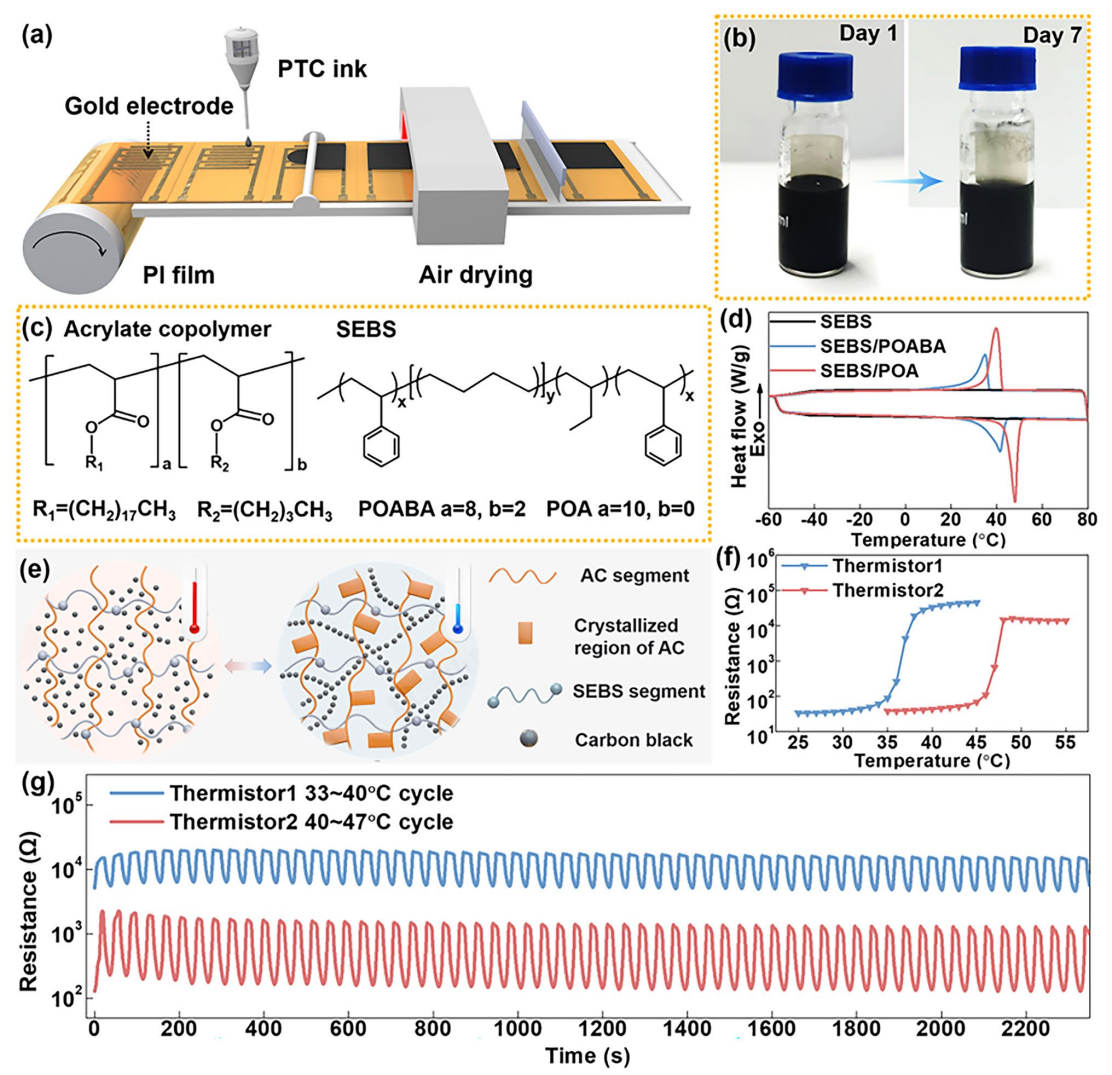
Preparation and thermo-response properties of PTC thermistors. **a** Preparation process of PTC thermistors. **b** The stability of PTC ink. **c** Molecular formula of functional polymers. **d** Melting and crystallization capacity of acrylate copolymers. **e** Working mechanism of PTC thermistors. **f** Temperature response performance of PTC thermistors. **g** The response stability of PTC thermistors

The process of sensing and feedback control of constant body temperature is attributed to the relaxation/contraction of capillaries under skin. To achieve self-regulation of temperature, a PTC thermosensitive ink was firstly prepared. The ink mainly composed of Styrene Ethylene Butylene Styrene (SEBS), acrylate copolymer (AC) and carbon black (CB) that dispersed in tetrahydrofuran (THF). Specifically, AC serves the purpose of temperature sensing, SEBS serves a toughening agent to maintain the shape stability above the melting point [42], and CB was acted as conductive component. As for fabrication process, the ink was firstly drop onto an electrode sheet to form a coating layer, after drying, the PTC thermistors can be obtained (Fig. 3a). The stability of the PTC ink is the key of the fabrication with its potential application. The ink has no significant stratification after being putting in a bottle and storing for a week, suggesting its long-term stability for the fabrication (Figs. 3b and S8). As the main component of the PTC thermistor, the AC is prepared according to our previous work, and the copolymerization process was happened between butyl acrylate (BA) and octadecyl acrylate (OA), as shown in Fig. 3c. Moreover, this copolymer with different switching temperatures can be obtained by adjusting the ratio of BA and OA [29, 43]. To realize the bidirectional control of the high and low physiological temperature, we selected POABA (OA/BA = 8/2) and POA (OA/BA = 10/0) as the thermistors. The microstructure of the PTC thermistor is shown in Fig. S9a, it can be seen that conductive carbon black is embedded in the polymer matrix. To explain the PTC effect is mainly related to the melting and crystallization capacity of AC. Differential scanning calorimetry (DSC) is carried out to analyze the melting and crystallization of AC (Fig. 3d), it is found that SEBS has no obvious melting and crystallization state, while the composite materials doped with POABA and POA show a huge melting peak at about 40 and 50 °C, respectively, indicating the melting and crystallization process of AC at 50 and 40 °C, respectively.

To explain the huge PTC effect of thermistors, we simulated the changes of the conductive path in the hybrid polymer system at different temperatures, as shown in Fig. 3e. Above the melting temperature, due to the high viscosity of the molten AC, the CB is uniformly dispersed in the whole polymer system, resulting in a large resistance. When the temperature drops below the crystallization temperature, the side chain of the AC begins to crystallize, the formation of the crystallization zone forces the CB to move to the amorphous zone, thus the conductive path forms and the resistance decreases. We further tested the temperature response of SEBS/CB (Fig. S9b), and the thermistors (Fig. 3f). Only the composites doped with AC show a huge PTC effect, and the switching temperature corresponds to the AC melting point. Therefore, the two thermistors can control the temperature between 35 and 45 °C. The biggest feature of the PTC thermistor is that there is a large resistance change near the switching temperature, and the response sensitivity is in various ways between different temperatures, as shown in Fig. S10a, b. According to different response characteristics of thermistors, T1 is expected to give feedback when cooling down, while T2 is working when heating up, as shown in Fig. S10c. Subsequently, we tested the repeatability of the thermistors (Fig. 3g). It can be seen that they showed excellent response stability and repeatability, which can be attributed to the repeatable side chain crystallization of AC and a certain limiting effect of the SEBS molecular chain [22]. As shown in Fig. S11, the response time of thermistors was investigated. It can be seen that the response time of T1 during heating is within 2 s, and the cooling time is within 8 s. Thermistor 2 from room temperature to 47 ºC, the response time during the heating process is within 2 s, and the cooling time is within 5 s.

### Circuit Design and Self-Regulating Performance of the TRES

**Fig. 4:**
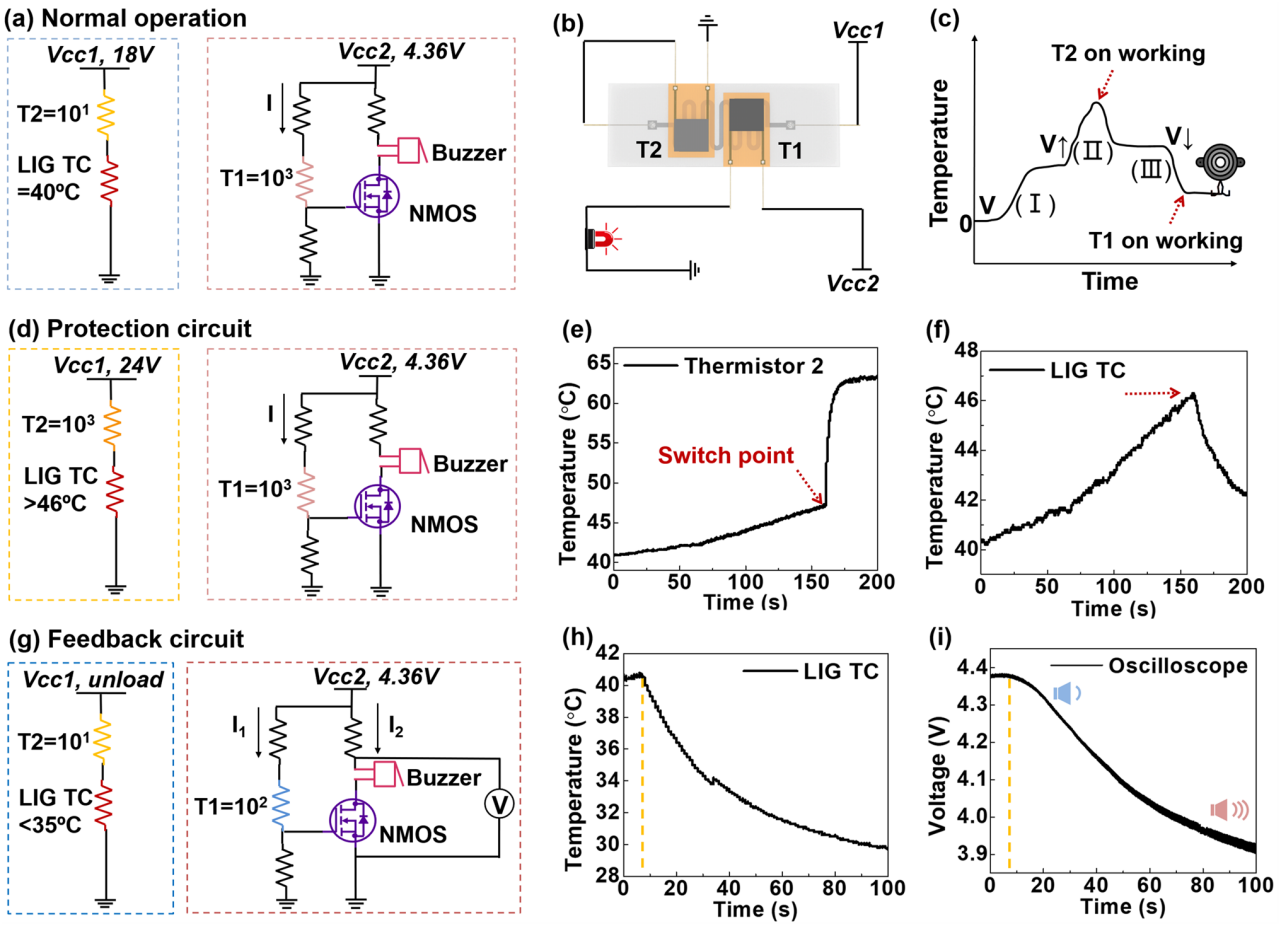
Circuit design and self-regulating performance of TRES. **a** Circuit diagram in normal working condition. **b** Schematic diagram of connection mode of protection circuit and feedback circuit. **c** Working mechanism of different thermistors. **d** Protection circuit diagram. **e** Real-time temperature during T2 working. **f** The temperature change of LIG TC on T2 working. **g** Feedback circuit diagram. **h** The temperature change of LIG TC on T1 working. **i** The indicator of oscilloscope changes in real time

In order to realize the self-regulating function of the TRES, a circuit was designed as shown in Fig. 4, which mainly consists of two parts: protection circuit and feedback circuit. First, the response of LIG TC combined with PTC thermistor was tested, as shown in Fig. S12a. We apply a 1 V bias voltage to the PTC thermistor and a different pulse voltage to the LIG TC achieving different temperature control. The results are shown in Fig. S12b, c, it can be seen that with pulse voltage increasing, the temperature of LIG TC keeps rising. The resistance of T1 changes abruptly between 15 and 20 V, while T2 resistance changes abruptly between 20 and 25 V. Corresponding to the relationship between voltage and temperature of LIG TC mentioned above, we have verified that switch temperature at T1 and T2 around 35 and 45 °C. As shown in Fig. 4a, b, the normal working state of the LIG TC and the two PTC thermistors was simulated by, which applying voltage to LIG TC until the temperature reached about 40 °C. As a result, T1 was in a high resistance state, and T2 was in a low resistance state. As the voltage continues to increase so that T2 exceeds its switching temperature of about 45 °C, T2 begins to become a high-resistance state, limiting the current passing through the LIG TC then its temperature drops. When the voltage suddenly decreases or is unloaded, and the temperature of LIG TC drops to T1’s switching temperature of about 35 °C, T1 begins to be in a low resistance state, activating the alarm in the other branch to warning. Figure 4c shows the working modes of T1 and T2.

A self-regulating circuit model based on the resistance change (∆R) of the thermistors was designed and verified. One part is protection circuit: LIG TC and T1 in series, Vcc1 adjustable. One part is feedback circuit: T2 and buzzer in parallel, Vcc2 = 4.36 V fixed (when T1 is low resistance, the minimum voltage value to activate the alarm). As shown in Fig. 4d, for the protection circuit, when Vcc1 increased to 18 V, LIG TC reached to temperature of 40 °C, and the resistance of T2 is only tens of Ω, so LIG TC obtains most of the voltage. On the outside circuit, T1 can reach a resistance of several thousand Ω, so R4 can only receive a small voltage, which cannot turn on the N-Metal–Oxide–Semiconductor (NMOS), thus the buzzer branch is blocked. When Vcc1 gradually increases to 24 V, it can be seen from Fig. 4e and f, that the temperature of T2 changes abruptly at around 160 s monitored by thermal imaging, at this time the temperature of LIG TC drops from 46 °C. This indicates that T1 provides protection to the LIG TC avoiding being too high. At this time, T1 is still in a high resistance state, and the buzzer branch is blocked. For the feedback circuit, as shown in Fig. 4g, a buzzer is used as an alarm. To further quantify the activation of the buzzer, we connected an oscilloscope at both ends of the buzzer and the NMOS as a voltmeter. When Vcc1 is suddenly unloaded, the temperature of LIG TC begins to drop rapidly (Fig. 4h), while the resistance of T1 also begins to decrease. When the voltage of R4 is large enough, the NMOS turn on and the buzzer starts ringing. With the temperature of LIG TC continuously decreasing, the NMOS gate voltage continuously increases, resulting in the current through the buzzer increasing, equal to the reduction voltage of the buzzer and NMOS. As shown in Fig. 4i, the oscilloscope records the process from the open circuit voltage to the buzzer getting louder, until the gate voltage stabilizes, means the temperature of LIG TC keeps constant. More detailed circuit information can be found in Fig. S13.

### TRES Promotes Wound Healing

**Fig. 5:**
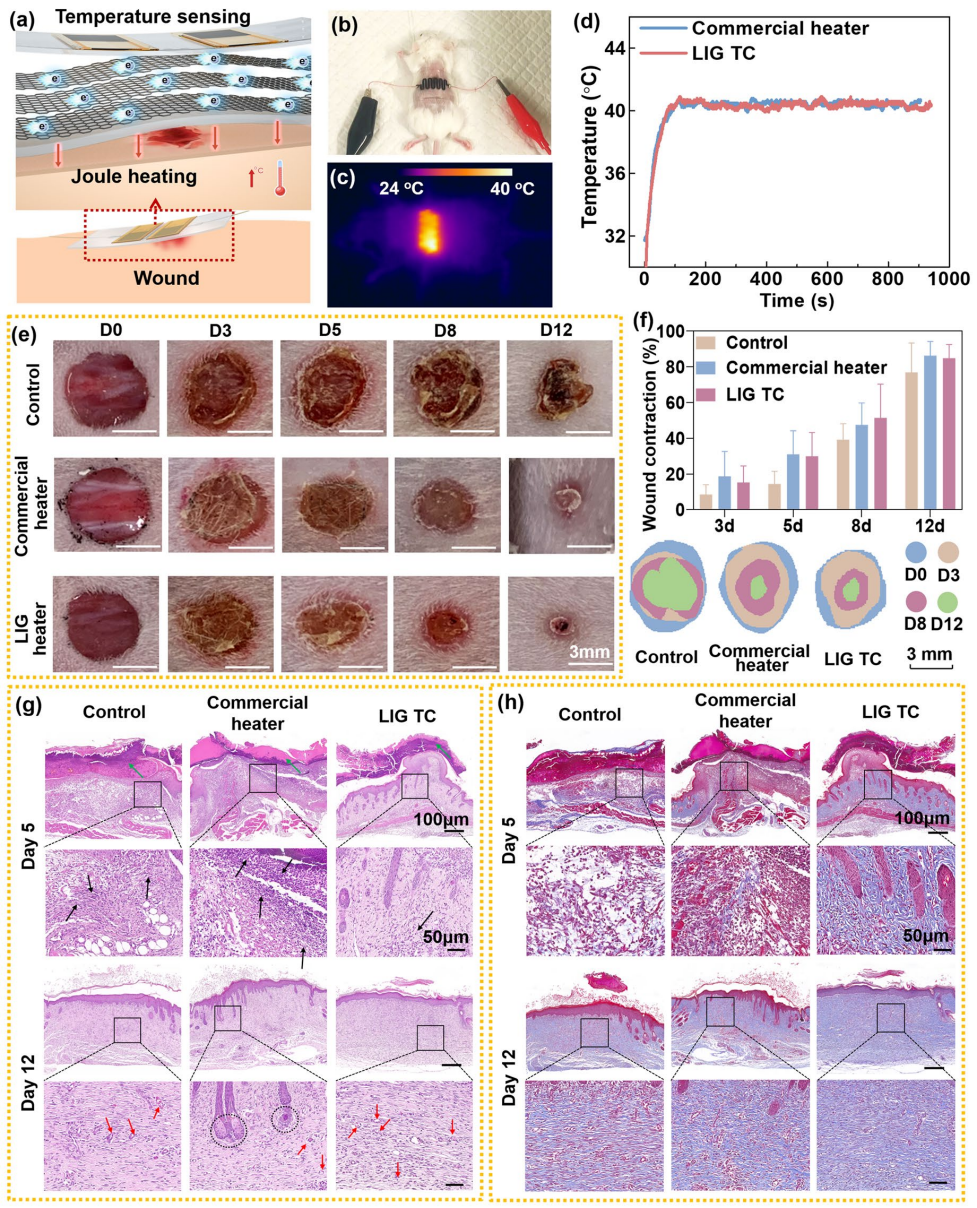
Evaluation of in vivo wound healing and histological analysis. **a** Illustruction of how TRES works in the wound. **b** Physical image of TRES placed on mice. **c** Thermal infrared imaging of mice heated by a TRES. **d** LIG TC and commercial heater monitor temperature in real-time. **e** Photographs of dorsal wounds of mice treated with nothing, commercial heater, and TRES. **f** The percentages of wound contraction in 12 days and the changes in the wound boundaries. **g, h** Histological analysis of the wounds treated via H&E staining and Masson staining (*n* = 3, *p* < 0.05)

In wounded skin, the lack of necessary thermos-sensitivity functions hindering the heal process of the wound. To this end, the skin-inspired TRES with both temperature monitoring and self-adjusting ability was applied to promote wound healing. The heating of the graphene array in this biomimetic skin is achieved by an external power supply device, which could increase the blood flow near the wound, effectively promoting wound healing and skin tissue repair, as shown in Fig. 5a [44, 45]. To demonstrate the positive effect of TRES on promoting wound healing, animal models with a notch on the back of mice were constructed. The effects of blank control group (control), commercial heater covering group, and TRES covering group were systematically studied on full-layer skin defect wound healing in mice. As one typical example, 40 °C was chose to demonstrate the promoting wound healing ability of biomimetic TRES.

As shown in Fig. 5b, c, similar to control samples, the TRES was placed at the wound area on the back of the mice. The temperature was monitored by a thermal imager (Fig. 5d). It can be seen that the response time and stability of the LIG TC were similar to the commercial heater. The trend of wound healing continued for 12 days during the entire observation period, as shown in Fig. 5e. It shows wound area of the TRES group and the commercial group decreased significantly, while the scab area of the BC group remained large for 12 days. The wound shrinkage rate was statistically mapped, as shown in Fig. 5f. The healing rate of the BC group reached 70% after 12 days, while the two heating groups can reach 80%, indicating significantly reduced the wound area.

To further evaluate the wound healing process, hematoxylin–eosin (H&E) staining and Masson tri-color staining were performed on day 5 and day 12, as shown in Fig. 5g, h. On day 5, more inflammatory cells (black arrows) were observed in the commercial group at the wound site, with serious inflammatory reactions. There were fewer inflammatory cells in the TRES group, and the re-epithelialization was better than in the commercial group. All three groups of wounds were covered with rod-shaped scabs (green arrows). More collagen fibers were generated in wound dermis, subcutaneous, and muscle tissues in the TRES group compared with that in the commercial group. On day 12, continuous epithelial tissue and dense connective tissue were formed in all three groups, and the inflammatory response was significantly relieved to varying degrees. Mature glandular tissue and capillaries appeared in the commercial group, and the number of new blood vessels in the TRES group was more than the other two groups. More collagen fibers were generated in wound dermis, subcutaneous, and muscle tissues in the TRES group than in the other two groups. Therefore, an appropriate increase in temperature can enhance tissue metabolism, promote re-epithelialization, angiogenesis, and collagen synthesis, and promote wound healing [46]. From the wound healing perspective, direct contact with biological tissue using inherently soft electronic materials with low Young’s modulus, such as TRES, can minimize adverse reactions [47].

## Conclusion

In summary, we have successfully fabricated an interactive flexible electronic system (TRES) by simulating the self-temperature regulation of human skin. LIG array was used as the temperature control unit, it has a high effective Joule thermal effect, that only 0.15 W power is needed to achieves to 40 °C. Two PTC thermistors are used as temperature regulating elements, which has order of magnitude resistance change near the switching temperature, and fast response time. Through the circuit design, TRES makes a cut-off for high temperature (46 °C) and a warning for low temperature (33 °C) to provide safe temperature protection for the human body. The device can also produce mild thermal stimulation that promotes wound healing with less inflammatory response than commercial heaters. the potential of TRES for temperature monitoring was also demonstrated, as shown in Fig. S17. Due to the large thermal expansion coefficient of PDMS (CTE = 15 × 10^−5^/K), the sensitivity of TRES is 4% at 25–45 °C, which is four times higher than that of PI substrate. Duo the high sensitivity, the TRES can distinguish 35, 36, and 37 °C accurately, indicating its potential in wound temperature monitoring. More importantly, TRES increased the wound healing rate by 10% compared to the control group, and effectively reduced the inflammatory response compared to the commercial heating group. The device we fabricated have great potential in the field of artificial skin and biomedical treatment devices.

## Supplementary Information

Below is the link to the electronic supplementary material.

Supplementary file1 (PDF 869 KB)

## References

[1] W.D. Li, K. Ke, J. Jia, J.H. Pu, X. Zhao et al., Recent advances in multiresponsive flexible sensors toward E-skin: a delicate design for versatile sensing. Small **18**, e2103734 (2022). 10.1002/smll.202103734 34825473 10.1002/smll.202103734

[2] M. Wang, Y. Luo, T. Wang, C. Wan, L. Pan et al., Artificial skin perception. Adv. Mater. **33**, 2003014 (2021). 10.1002/adma.202003014 10.1002/adma.202003014 32930454

[3] J.Y. Oh, Z. Bao, Second skin enabled by advanced electronics. Adv. Sci. **6**, 1900186 (2019). 10.1002/advs.201900186 10.1002/advs.201900186 PMC6548954 31179225

[4] A. Chortos, J. Liu, Z. Bao, Pursuing prosthetic electronic skin. Nat. Mater. **15**, 937–950 (2016). 10.1038/nmat4671 27376685 10.1038/nmat4671

[5] C. Wang, C. Pan, Z. Wang, Electronic skin for closed-loop systems. ACS Nano **13**, 12287–12293 (2019). 10.1021/acsnano.9b06576 31626533 10.1021/acsnano.9b06576

[6] L.E. Osborn, R. Venkatasubramanian, M. Himmtann, C.W. Moran, J.M. Pierce et al., Evoking natural thermal perceptions using a thin-film thermoelectric device with high cooling power density and speed. Nat. Biomed. Eng. (2023). 10.1038/s41551-023-01070-w 10.1038/s41551-023-01070-w 37500749

[7] S.R. Madhvapathy, J.J. Wang, H. Wang, M. Patel, A. Chang et al., Implantable bioelectronic systems for early detection of kidney transplant rejection. Science **381**, 1105–1112 (2023). 10.1126/science.adh7726 37676965 10.1126/science.adh7726

[8] M. Jiang, Q. Shen, J. Zhang, S. An, S. Ma et al., Bioinspired temperature regulation in interfacial evaporation. Adv. Funct. Mater. **30**, 1910481 (2020). 10.1002/adfm.201910481

[9] C.L. Tan, E.K. Cooke, D.E. Leib, Y.C. Lin, G.E. Daly et al., Warm-sensitive neurons that control body temperature. Cell **167**, 47–59 (2016). 10.1016/j.cell.2016.08.028 27616062 10.1016/j.cell.2016.08.028 PMC5062957

[10] R. Dong, B. Guo, Smart wound dressings for wound healing. Nano Today **41**, 101290 (2021). 10.1016/j.nantod.2021.101290

[11] D. Lou, Q. Pang, X. Pei, S. Dong, S. Li et al., Flexible wound healing system for pro-regeneration, temperature monitoring and infection early warning. Biosens. Bioelectron. **162**, 112275 (2020). 10.1016/j.bios.2020.112275 32392156 10.1016/j.bios.2020.112275

[12] P. Tang, Y. Liu, Y. Liu, H. Meng, Z. Liu et al., Thermochromism-induced temperature self-regulation and alternating photothermal nanohelix clusters for synergistic tumor chemo/photothermal therapy. Biomaterials **188**, 12–23 (2019). 10.1016/j.biomaterials.2018.10.008 30317112 10.1016/j.biomaterials.2018.10.008

[13] Y. Gao, H. Du, Z. Xie, M. Li, J. Zhu et al., Self-adhesive photothermal hydrogel films for solar-light assisted wound healing. J. Mater. Chem. B **7**, 3644–3651 (2019). 10.1039/C9TB00481E

[14] G. Chen, K. Hou, N. Yu, P. Wei, T. Chen et al., Temperature-adaptive hydrogel optical waveguide with soft tissue-affinity for thermal regulated interventional photomedicine. Nat. Commun. **13**, 7789 (2022). 10.1038/s41467-022-35440-w 36526631 10.1038/s41467-022-35440-w PMC9758120

[15] S. Zhao, R. Zhu, Electronic skin with multifunction sensors based on thermosensation. Adv. Mater. **29**, 1606151 (2017). 10.1002/adma.201606151 10.1002/adma.201606151 28195430

[16] Y. Lee, J. Park, A. Choe, S. Cho, J. Kim et al., Mimicking human and biological skins for multifunctional skin electronics. Adv. Funct. Mater. **30**, 1904523 (2020). 10.1002/adfm.201904523

[17] K. Kwon, J.U. Kim, S.M. Won, J. Zhao, R. Avila et al., A battery-less wireless implant for the continuous monitoring of vascular pressure, flow rate and temperature. Nat. Biomed. Eng. **7**, 1215–1228 (2023). 10.1038/s41551-023-01022-4 37037964 10.1038/s41551-023-01022-4

[18] W. Ouyang, W. Lu, Y. Zhang, Y. Liu, J.U. Kim et al., A wireless and battery-less implant for multimodal closed-loop neuromodulation in small animals. Nat. Biomed. Eng. **7**, 1252–1269 (2023). 10.1038/s41551-023-01029-x 37106153 10.1038/s41551-023-01029-x

[19] J. Tu, J. Min, Y. Song, C. Xu, J. Li et al., A wireless patch for the monitoring of C-reactive protein in sweat. Nat. Biomed. Eng. **7**, 1293–1306 (2023). 10.1038/s41551-023-01059-5 37349389 10.1038/s41551-023-01059-5 PMC10592261

[20] S. Kim, Y.S. Oh, K. Lee, S. Kim, W.-Y. Maeng et al., Battery-free, wireless, cuff-type, multimodal physical sensor for continuous temperature and strain monitoring of nerve. Small **19**, 2206839 (2023). 10.1002/smll.202206839 10.1002/smll.202206839 37069777

[21] R. Chen, T. Luo, D. Geng, Z. Shen, W. Zhou, Facile fabrication of a fast-response flexible temperature sensor via laser reduced graphene oxide for contactless human-machine interface. Carbon **187**, 35–46 (2022). 10.1016/j.carbon.2021.10.064

[22] C. Okutani, T. Yokota, T. Someya, Ultrathin fiber-mesh polymer thermistors. Adv. Sci. **9**, e2202312 (2022). 10.1002/advs.202202312 10.1002/advs.202202312 PMC9596841 36057993

[23] C. Okutani, T. Yokota, R. Matsukawa, T. Someya, Suppressing the negative temperature coefficient effect of resistance in polymer composites with positive temperature coefficients of resistance by coating with parylene. J. Mater. Chem. C **8**, 7304–7308 (2020). 10.1039/D0TC00702A

[24] M. Sang, K. Kang, Y. Zhang, H. Zhang, K. Kim et al., Ultrahigh sensitive Au-doped silicon nanomembrane based wearable sensor arrays for continuous skin temperature monitoring with high precision. Adv. Mater. **34**, e2105865 (2022). 10.1002/adma.202105865 34750868 10.1002/adma.202105865

[25] G.Y. Bae, J.T. Han, G. Lee, S. Lee, S.W. Kim et al., Pressure/temperature sensing bimodal electronic skin with stimulus discriminability and linear sensitivity. Adv. Mater. **30**, e1803388 (2018). 10.1002/adma.201803388 30216564 10.1002/adma.201803388

[26] M. Li, Y. Shi, H. Gao, Z. Chen, Bio-inspired nanospiky metal particles enable thin, flexible, and thermo-responsive polymer nanocomposites for thermal regulation. Adv. Funct. Mater. **30**, 1910328 (2020). 10.1002/adfm.201910328

[27] M. Li, G. Cai, J. Holoubek, K. Yu, H. Liu et al., Hierarchically structured metal carbides as conductive fillers in thermo-responsive polymer nanocomposites for battery safety. Nano Energy **103**, 107726 (2022). 10.1016/j.nanoen.2022.107726

[28] Z. Chen, P.-C. Hsu, J. Lopez, Y. Li, J.W.F. To et al., Fast and reversible thermoresponsive polymer switching materials for safer batteries. Nat. Energy **1**, 15009 (2016). 10.1038/nenergy.2015.9

[29] T. Yokota, Y. Inoue, Y. Terakawa, J. Reeder, M. Kaltenbrunner et al., Ultraflexible, large-area, physiological temperature sensors for multipoint measurements. Proc. Natl. Acad. Sci. U.S.A. **112**, 14533–14538 (2015). 10.1073/pnas.1515650112 26554008 10.1073/pnas.1515650112 PMC4664374

[30] M. Li, K. Chang, W. Zhong, C. Xiang, W. Wang et al., A highly stretchable, breathable and thermoregulatory electronic skin based on the polyolefin elastomer nanofiber membrane. Appl. Surf. Sci. **486**, 249–256 (2019). 10.1016/j.apsusc.2019.04.271

[31] S. Xiang, D. Liu, C. Jiang, W. Zhou, D. Ling et al., Liquid-metal-based dynamic thermoregulating and self-powered electronic skin. Adv. Funct. Mater. **31**, 2100940 (2021). 10.1002/adfm.202100940

[32] S. Xiang, J. Tang, L. Yang, Y. Guo, Z. Zhao et al., Deep learning-enabled real-time personal handwriting electronic skin with dynamic thermoregulating ability. npj Flex. Electron. **6**, 59 (2022). 10.1038/s41528-022-00195-3

[33] J. Huang, Z. Xu, W. Qiu, F. Chen, Z. Meng et al., Stretchable and heat-resistant protein-based electronic skin for human thermoregulation. Adv. Funct. Mater. **30**, 1910547 (2020). 10.1002/adfm.201910547

[34] J. Wu, W. Huang, Y. Liang, Z. Wu, B. Zhong et al., Self-calibrated, sensitive, and flexible temperature sensor based on 3D chemically modified graphene hydrogel. Adv. Electron. Mater. **7**, 2001084 (2021). 10.1002/aelm.202001084

[35] R. You, Y.-Q. Liu, Y.-L. Hao, D.-D. Han, Y.-L. Zhang et al., Laser fabrication of graphene-based flexible electronics. Adv. Mater. **32**, 1901981 (2020). 10.1002/adma.201901981 10.1002/adma.201901981 31441164

[36] T.S.D. Le, H.P. Phan, S. Kwon, S. Park, Y. Jung et al., Recent advances in laser-induced graphene: mechanism, fabrication, properties, and applications in flexible electronics. Adv. Funct. Mater. **32**, 2205158 (2022). 10.1002/adfm.202205158

[37] G. Karimi, I. Lau, M. Fowler, M. Pope, Parametric study of laser-induced graphene conductive traces and their application as flexible heaters. Int. J. Energy Res. **45**, 13712–13725 (2021). 10.1002/er.6701

[38] Z. Sun, S. Fang, Y.H. Hu, 3D graphene materials: from understanding to design and synthesis control. Chem. Rev. **120**, 10336–10453 (2020). 10.1021/acs.chemrev.0c00083 32852197 10.1021/acs.chemrev.0c00083

[39] J. Xu, R. Li, S. Ji, B. Zhao, T. Cui et al., Multifunctional graphene microstructures inspired by honeycomb for ultrahigh performance electromagnetic interference shielding and wearable applications. ACS Nano **15**, 8907–8918 (2021). 10.1021/acsnano.1c01552 33881822 10.1021/acsnano.1c01552

[40] Y. Qiao, Y. Wang, H. Tian, M. Li, J. Jian et al., Multilayer graphene epidermal electronic skin. ACS Nano **12**, 8839–8846 (2018). 10.1021/acsnano.8b02162 30040381 10.1021/acsnano.8b02162

[41] S.Y. Xia, Y. Long, Z. Huang, Y. Zi, L.Q. Tao et al., Laser-induced graphene (LIG)-based pressure sensor and triboelectric nanogenerator toward high-performance self-powered measurement-control combined system. Nano Energy **96**, 107099 (2022). 10.1016/j.nanoen.2022.107099

[42] J. Jeon, H.-B.-R. Lee, Z. Bao, Flexible wireless temperature sensors based on Ni microparticle-filled binary polymer composites. Adv. Mater. **25**, 850–855 (2013). 10.1002/adma.201204082 23233317 10.1002/adma.201204082

[43] Y. Geng, R. Cao, M.T. Innocent, Z. Hu, L. Zhu et al., A high-sensitive wearable sensor based on conductive polymer composites for body temperature monitoring. Compos. Part A Appl. Sci. Manuf. **163**, 107269 (2022). 10.1016/j.compositesa.2022.107269

[44] L. Liu, R. Li, F. Liu, L. Huang, W. Liu et al., Highly elastic and strain sensing corn protein electrospun fibers for monitoring of wound healing. ACS Nano **17**, 9600–9610 (2023). 10.1021/acsnano.3c03087 37130310 10.1021/acsnano.3c03087

[45] X. Tang, X. Chen, S. Zhang, X. Gu, R. Wu et al., Silk-inspired *in situ* hydrogel with anti-tumor immunity enhanced photodynamic therapy for melanoma and infected wound healing. Adv. Funct. Mater. **31**, 2101320 (2021). 10.1002/adfm.202101320

[46] X. Xu, X. Liu, L. Tan, Z. Cui, X. Yang et al., Controlled-temperature photothermal and oxidative bacteria killing and acceleration of wound healing by polydopamine-assisted Au-hydroxyapatite nanorods. Acta Biomater. **77**, 352–364 (2018). 10.1016/j.actbio.2018.07.030 30030176 10.1016/j.actbio.2018.07.030

[47] T. Someya, Z. Bao, G.G. Malliaras, The Rise of plastic bioelectronics. Nature **540**, 379–385 (2016). 10.1038/nature21004 27974769 10.1038/nature21004

