# Supplementary material for: A Skin-Inspired Self-Adaptive System for Temperature Control During Dynamic Wound Healing
Source: Nanomicro Lett. 2024 Mar 11;16:152. doi: 10.1007/s40820-024-01345-0 (PMC10928041; doi:10.1007/s40820-024-01345-0)
Supplement: Supplementary file 1 — Supplementary file1 (PDF 869 KB) [file 40820_2024_1345_MOESM1_ESM.pdf]

Supporting Information for

A Skin-Inspired Self-Adaptive System for Temperature Control During Dynamic Wound Healing

Yaqi Geng[1,#] Guoyin Chen[1,#] Ran Cao[1,*] Hongmei Dai[1], Zexu Hu[1], Senlong Yu[1], Le Wang[1], Liping Zhu[1], Hengxue Xiang[1,*], Meifang Zhu[1,*]

[1]State Key Laboratory for Modification of Chemical Fibers and Polymer Materials, College of Materials Science and Engineering, Donghua University, 2999 North Renmin Road, Shanghai 201620, P. R. China

[#]Yaqi Geng and Guoyin Chen contributed equally to this article.

*Corresponding authors. E-mail: rancao@dhu.edu.cn (Ran Cao), hengxuexiang@dhu.edu.cn (Hengxue Xiang), zhumf@dhu.edu.cn (Meifang Zhu)

Supplementary Figures

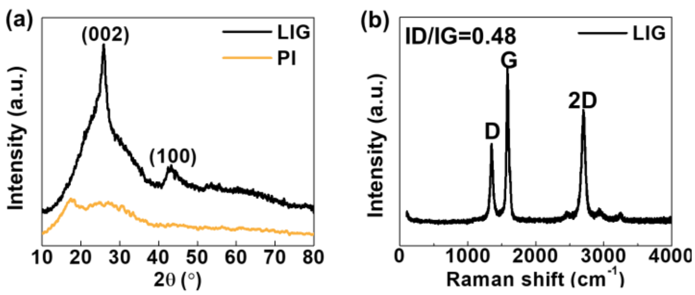

Fig. S1 (**a**) The crystallization analysis of LIG and PI (XRD). (**b**) Raman spectroscopy of LIG. (**c**) FTIR spectrogram analysis of LIG and PI

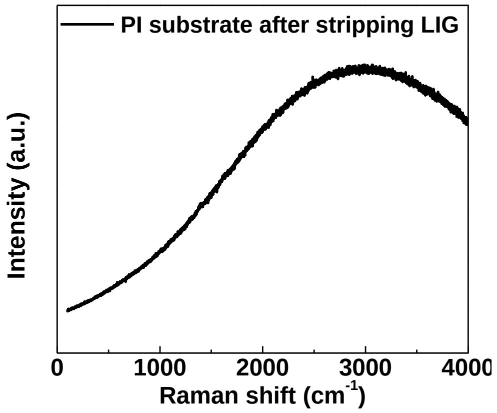

Fig. S2 Raman spectroscopy of PI substrate after stripping LIG

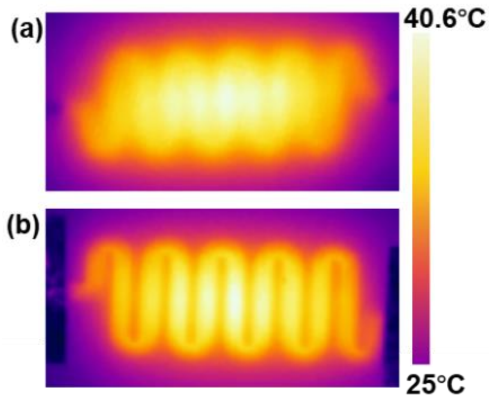

Fig. S3 (**a**) LIG TC in this work. (**b**) LIG on the PI substrate. (before transferred)

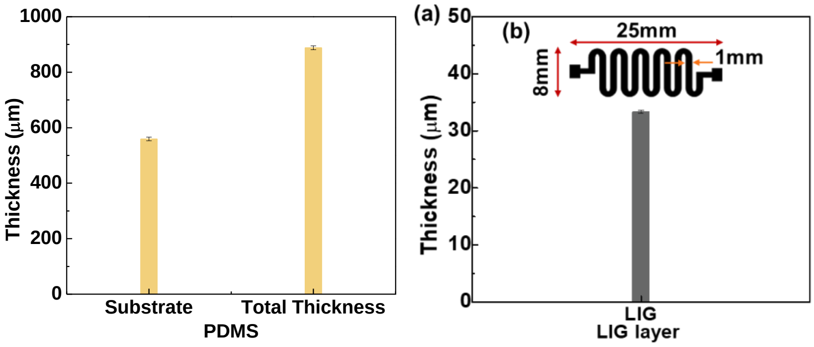

Fig. S4 (**a**) The thickness of LIG. (**b**) The size of LIG. (**c**) The thickness of PDMS

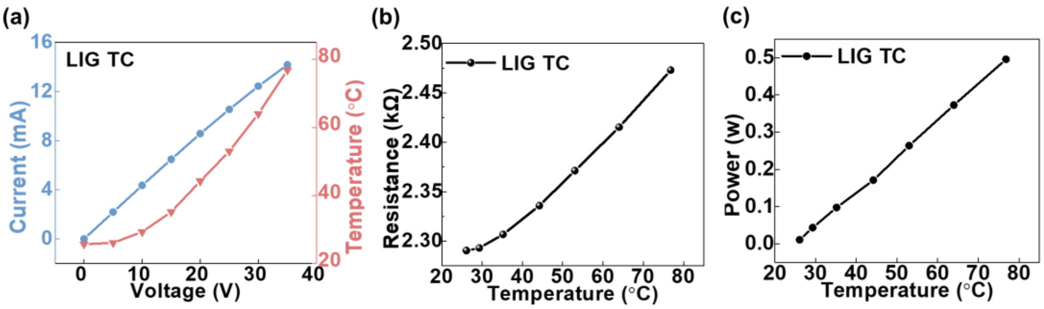

Fig. S5 (**a**) The relation of temperature and current of LIG TC with voltage. (**b**) The relationship between temperature and resistance of LIG TC. (**c**) The relationship between temperature and power of LIG TC

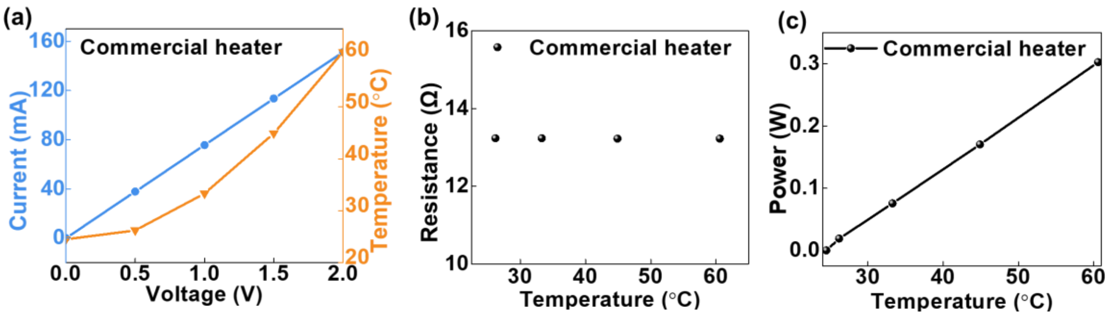

Fig. S6 (**a**) The relation of temperature and current of commercial heater with voltage. (**b**) The relationship between temperature and resistance of commercial heater. (**c**) The relationship between temperature and power of commercial heater

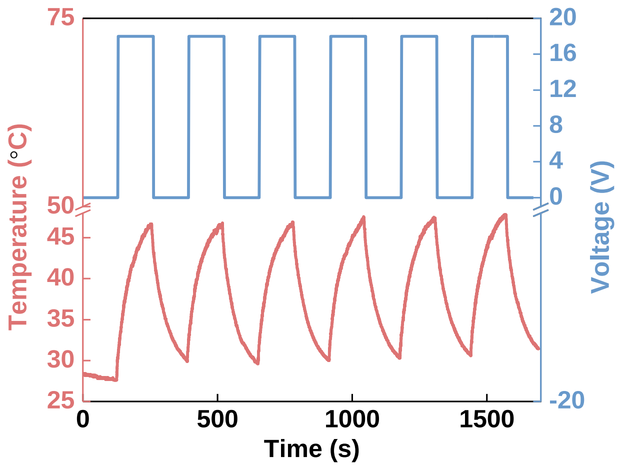

Fig. S7 Heating cycle stability of LIG TC

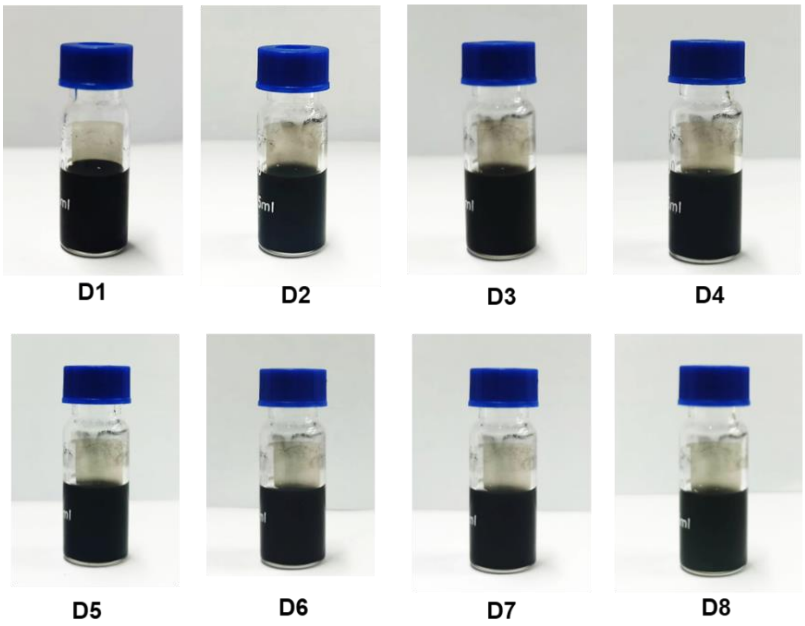

Fig. S8 The stability of PTC ink (a week)

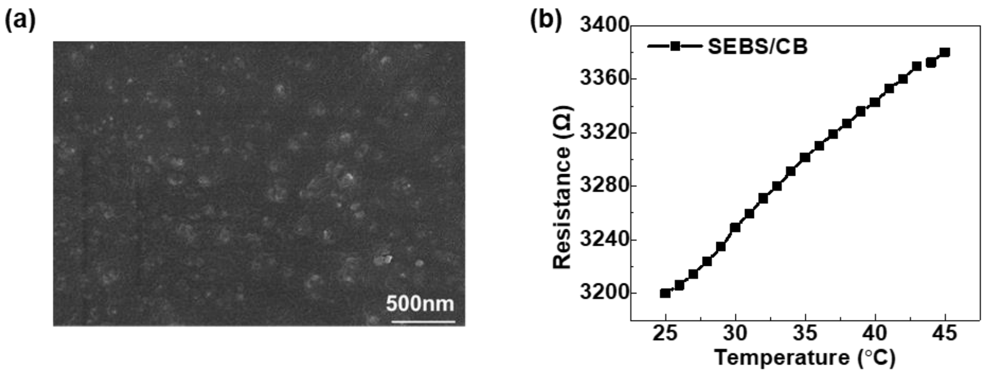

Fig. S9 (**a**) The SEM image of SEBS/AC/CB. (**b**) Temperature response performance of SEBS/CB

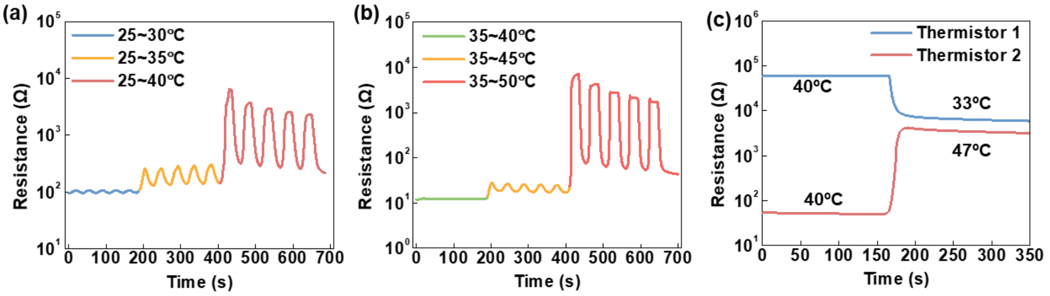

Fig. S10 (**a**) Temperature response performance of thermistor 1. (**b**) Temperature response performance of thermistor 2. (**c**) The sensitivity of thermistors 1 and 2 to the target temperature range

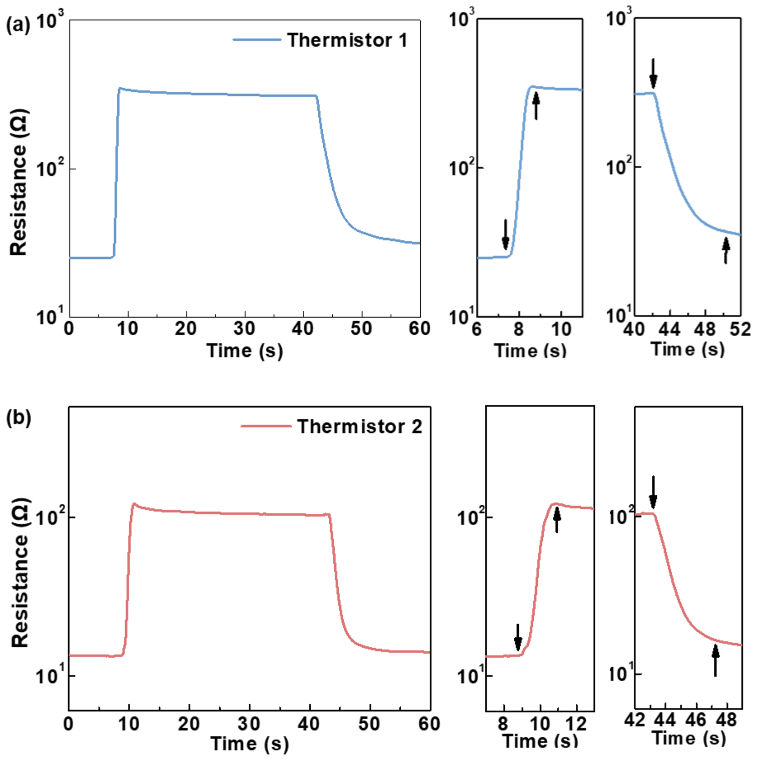

Fig. S11 (**a**) Response time of thermistor 1 from room temperature to 40 ºC. (**b**) Response time of thermistor 2 from room temperature to 47 ºC

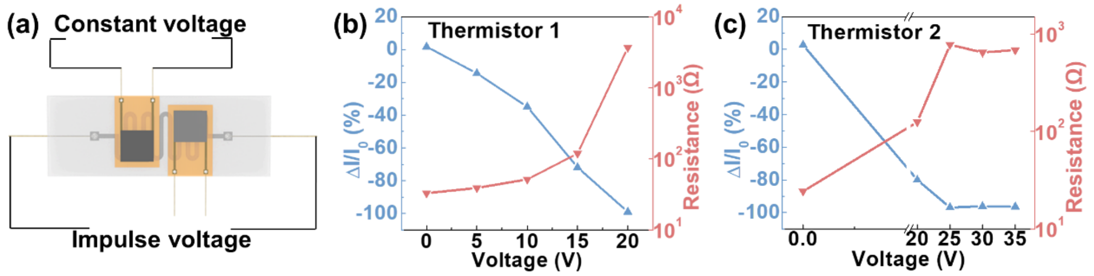

Fig. S12 (**a**) The connection method for testing TRES sensitivity. (**b**) The response of T1 by applying different voltages to the LIG TC. (**c**) The response of T2 by applying different voltages to the LIG TC

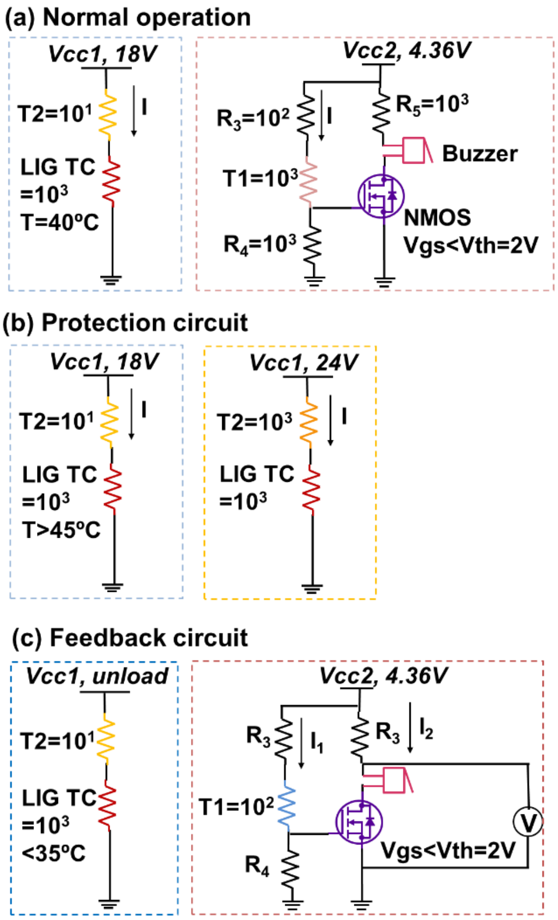

Dc power supply: DPS605U (60V5A); Joule heat element: LIG TC; Thermistors: T1 (switching temperature 35 ºC); T2 (switching temperature 45 ºC); Fixed resistance: R3=102, R4=103, R5=103; Alarm: Buzzer SFM-27, 3000Hz; Switching element: N channel, MOSFET, IRF530NPBF/TO-220

Fig. S13 Circuit details

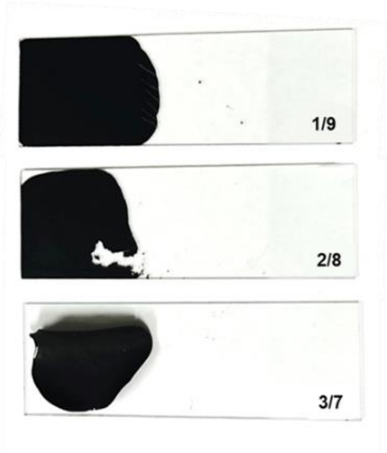

Fig. S14 Toughening effect of composites with increasing SEBS proportion

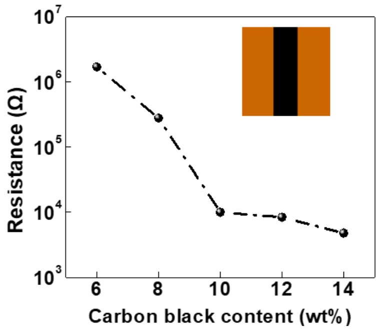

Fig. S15 Seepage threshold of the composite

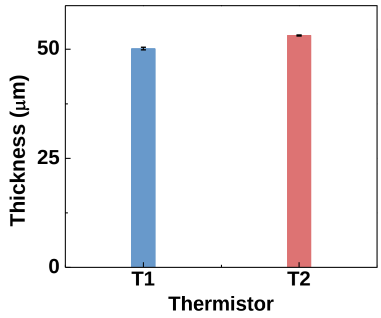

Fig. S16 Thickness of the thermistors

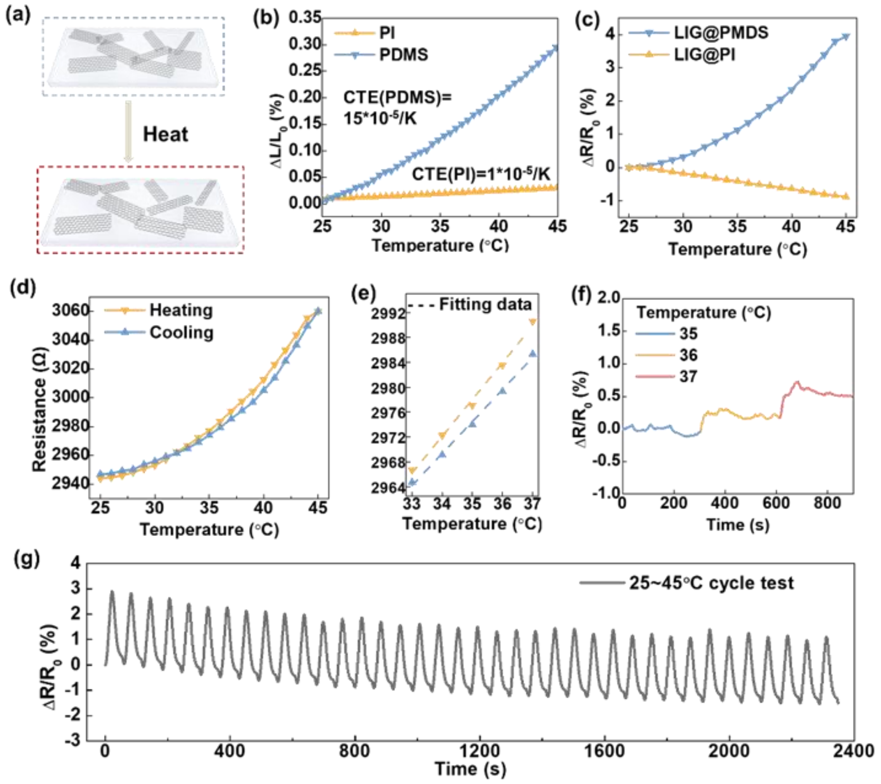

Fig. S17 (**a**) Thermal response mechanism of TRES. (**b**) Thermal expansion coefficient of PDMS and PI. (**c**) The temperature response sensitivity of LIG on different substrates. (**d**) The temperature response of LIG@PDMS between 25 and 45 °C. (**e**) LIG@PDMS has a near linear response in 33-37 °C. (**f**) Temperature response sensitivity of LIG@PDMS near body temperature. (**g**) The response cyclic stability of LIG@PDMS between 25 and 45 °C
